# Fluorescent angioscopic imaging of calcium phosphate tribasic: precursor of hydroxyapatite, the major calcium deposit in human coronary plaques

**DOI:** 10.1007/s10554-017-1142-y

**Published:** 2017-04-21

**Authors:** Takanobu Kobayashi, Osamu Nakagawa, Seiichiro Shirai, Ei Shimoyama, Nobuyuki Hiruta, Yasumi Uchida

**Affiliations:** 1 Department of Cardiology, Chiba-Kensei Hospital, Chiba, Japan; 2 Department of Cardiology, Funabashi-Futawa Hospital, Funabashi, Japan; 3 Department of Pathology, Funabashi-Futawa Hospital, Funabashi, Japan; 4 Department of Pathology, Toho University Medical Center Sakura Hospital, Sakura, Japan; 5 Japan Foundation for Cardiovascular Research, Funabashi, Japan

**Keywords:** Calcium phosphate tribasic, Color fluorescent angioscopy, Human coronary plaques, Lac dye

## Abstract

Coronary calcification is a risk factor for ischemic heart disease. Hydroxyapatite that is formed by polymerization from calcium phosphate tribasic (CPT) is the major constituent of coronary calcium deposits. If CPT could be visualized, coronary calcification could be predicted and prevented. We discovered that when CPT and collagen I, the main constituent of collagen fibers, are mixed with lac dye (LD) and then exposed to fluorescent light excited at 345 ± 15 nm and emitted at 420 nm, a purple fluorescence that is characteristic of CPT only is elicited. So, we examined localization of CPT and its relation to plaque morphology by color fluorescent angioscopy (CFA) or microscopy (CFM) in 24 coronary arteries obtained from 12 autopsy subjects. By CFA, the incidence (%) of CPT as confirmed by purple fluorescence in 15 normal segments, 25 white plaques, 14 yellow plaques without necrotic core (NC) and 8 yellow plaques with NC was 20, 36, 64 and 100 (p < 0.05 vs. normal segments), respectively. By CFM, the CPT was either deposited alone amorphously or surrounded hydroxyapatite that was identified by Oil Red O, methylene blue and von Kossa’s stain. The results suggested that CFA using LD is feasible for imaging CPT, that is a precursor of hydroxyapatite, in human coronary plaques, and this technique would help prediction and discovery of a preventive method of coronary calcification.

**Electronic supplementary material:**

The online version of this article (doi:10.1007/s10554-017-1142-y) contains supplementary material, which is available to authorized users.

## Introduction

Vascular calcification is known as a risk factor for cardiovascular diseases [1, 2], and of the at least 11 calcium compounds that can deposit in the human vascular wall. In these compounds, hydroxyapatite [Ca_5_(OH)(PO_4_)_3_]_x_ is the major constituent, and which is formed by polymerization from calcium phosphate tribasic [Ca_5_(OH)(PO_4_)_3_] (CPT) [3, 4]. So, if CPT could be visualized, vascular calcification could be predicted and preventive therapy for formation of hydroxyapatite by inhibiting polymerization could be invented. For this purpose, it is necessary whether and how CPT is deposited in human vascular wall, but the localization of CPT in the human vascular wall is poorly understood because there are currently no clinically applicable techniques to detect it in vivo.

Because we had discovered that lac dye (LD), which is used as a food coloring [5] and has an inhibitory action on carcinoma [6], elicits a fluorescence characteristic of CPT only, we aimed to use it as a biomarker in the present study of the localization of CPT in ex vivo human coronary plaques by color fluorescent angioscopy (CFA) [7–10] or microscopy (CFM) [9].

## Methods

### Detection of fluorescence characteristic of CPT by CFM

A CFM system with a band pass filter (BPF) of 340 ± 15 nm and a band absorption filter (BAF) of 420 nm was used for fluorescent imaging. The details of CFM are described elsewhere [7–9]. The intensity of fluorescence was categorized as strong, weak or absent when the exposure time required for imaging was ≤1, between 1 and 5 s, and >5 s, respectively.

LD (Wako Co., Osaka, Japan) was diluted in distilled water to a concentration of 10^−5^ M (the maximum concentration that does not precipitate) at 37 °C and then mixed with each of the major substances that comprise atherosclerotic plaques [8] with and without collagen I that mainly constitutes vascular intima. The evoked fluorescence was photographed at ×40.

Further, it was examined whether other 10 calcium compounds {i.e., hydroxyapatite, calcium phosphate dibasic (CaHPO_4_·2H_2_O), α-, β- tri-calcium phosphate [Ca_3_(PO_4_)_2_], calcium pyrophosphate [Ca_2_(PO_3_)(PO_4_)], calcium oxalate monohydrate (CaC_2_O_4_·H_2_O), calcium carbonate (CaCO_3_), calcium hydroxide [Ca(OH)_2_], calcium sulfide (Ca_2_SO_4_), and calcium chloride (CaCl_2_)} exhibit the same fluorescent color as CPT in the presence of LD.

### CFA system

The CFA system consisted of a fluorescence excitation unit with a band-pass filter (BPF) of 345 ± 15 nm, an angioscope (VecMover, Clinical Supply Co, Gifu, Japan), a fluorescence emission unit with a band -absorption filter (BAF) of 420 nm and a camera. The system has been approved for clinical use by the Japanese Ministry of Health and Labor, supported by National Insurance, on a commercial basis in Japan [10]. The intensity of the fluorescence images was arbitrarily defined as strong, weak and absent when the exposure-time required for imaging was ≤1 s, between1 and 5 s, and >5 s, respectively.

### Conventional angioscopy (CA) system

The CA system is consisted of an angioscope used for CFA, light source and a 3-coupled chilled device digital camera (Clinical Supply Co). The details of the procedure are described elsewhere [10].

### Definition of coronary plaques by CA

Plaque by CA was defined as a nonmotile and protruding or lining mass clearly demarcated from the adjacent normal wall and whose shape, location and color did not alter after saline solution flush. Plaques were further classified as white or yellow based on their surface color. A normal segment was defined as a milky white and smooth-surfaced portion of the vessel without any protrusion [8–12]. Surface color of the plaques was measured by an AquaCosmos image analyzer (C7746, Hamamatsu Photonics, Hamamatsu, Japan) [8].

### Angioscopic study on coronary plaques obtained from autopsy subjects

#### Ethics statement

The ex vivo study of coronary artery obtained from autopsy cases was carried out with the approval of the ethical committees of the Japan Foundation for Cardiovascular Research, Funabashi-Futawa Hospital, Chiba-kensei Hospital and Toho University, and after obtaining written informed consent from the families concerned.

#### Subjects

From April 1, 2015 to June 30, 2016, 24 proximal to middle segments of coronary arteries 5–12 cm in length (12 left anterior descending arteries and 12 left circumflex arteries) were excised from 12 successive autopsy cases [age 62 ± 6 (mean ± SD) years, 5 females, 7 males; cause of death: acute myocardial infarction (2), diabetes mellitus (1), chronic renal disease (2), hepatocellular carcinoma (1) cerebral infarction (2), pneumonia (2), gastric cancer (1), pancreatic cancer (1),] within 6–12 h after death. The experimental studies were performed 4–7 h later.

#### CA and CFA procedure

**Fig. 1:**
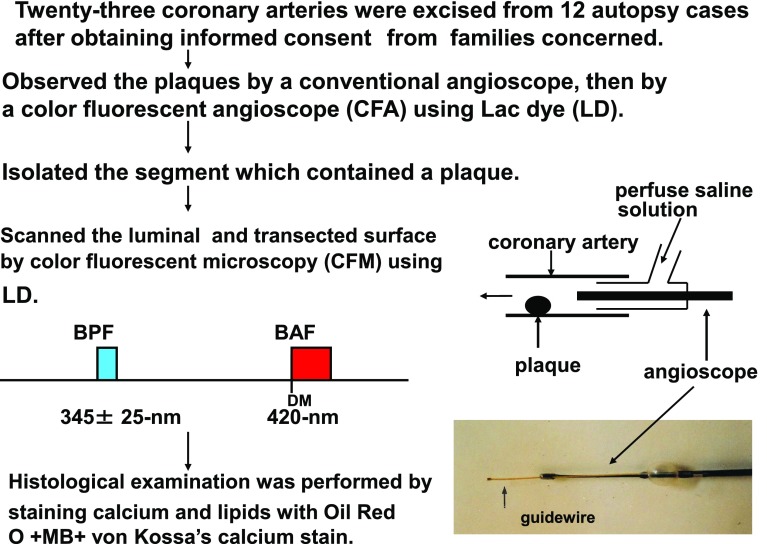
Schematic representation of experimental procedures *BPF* band-pass filter, *BAF* band-absorption filter

CA was performed as follows: A Y-connector was introduced into the proximal portion of each coronary artery for perfusion with saline solution at a rate of 10 mL/min and then the angioscope was introduced through the connector for observation of the artery. Initially, CA was carried out to detect plaque and because the light irradiated from the angioscope tip was visible through the coronary wall, the location of the target plaque could be confirmed (Fig. 1).

After observation by CA, the light guide and the image guide were connected to the fluorescence excitation and emission units, respectively. The BPF and BAF were set and a control image was obtained under the perfusion with saline solution. After ceasing the perfusion, 0.5 mL of 2% LD solution was injected into the perfusion circuit and 5 min later, saline perfusion was restarted and the target plaque was imaged again. Angioscopic images were analyzed by two independent observers who did not participate in the angioscopic studies.

### CFM study

A total of 47 plaques were confirmed by CA in 24 arteries. The 4–5 mm-long portion of vessel in which the observed plaque was located was isolated by transecting the proximal and the distal ends at the shorter axes to avoid any damage to the plaque. Subsequently, the isolated segment was cut longitudinally to open the lumen. The 15 normal segments were similarly isolated. The 47 isolated coronary segments that contained plaques and the 15 normal segments, were each mounted on a deck glass in such a way that the luminal surface of the plaque faced the deck glass. The surface was then scanned by CFM at ×10 or ×40 magnification using light wavelength filters in similar to those used for CFA.

The 47 plaques were transected across the center and half was again immersed in LD solution for 5 min to ensure penetration of the entire wall. It was mounted as before on a deck glass and the transected surface was scanned by CFM to examine localization of CPT, intimal thickness and the presence or absence of a necrotic core (NC). The angioscopically classified normal segments was reclassified as white plaque when their intimal thickness exceeded 300 µm. The angioscopically classified yellow plaques were classified as those with NC or without NC.

After CFM scanning, the adjacent raw sample, which was cut into slices of 30–40 μm thickness along the shorter axis, was stained with von Kossa’s calcium staining followed by Oil Red O and methylene blue (MB) for histological study using conventional microscopy. By this staining, calcium compounds were stained purple, dark brown or black, lipids as red, and collagen fibers and smooth muscles as blue. Necrotic core is a clearly demarcated glue-like portion which contains lipid deposits and debris stained red and devoid of collagen fibers, and because in liquid form they are often lost during staining. In our preliminary experiments, CPT was stained purple, calcium pyrophosphate and hydroxide black, and hydroxyapatite dark brown, but the remaining calcium compounds were not stained.

### Statistical analysis

The data obtained were expressed as mean ± standard deviation (SD), and tested by Fisher’s exact test or Mann–Whitney’s U-test. A value of p < 0.05 was considered to be statistically significant.

## Results

### Color fluorescence of CPT

**Fig. 2:**
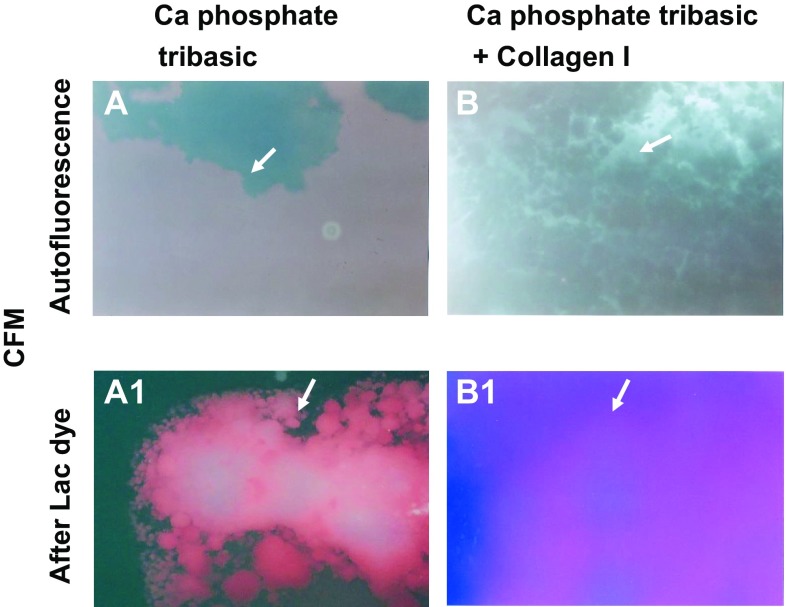
Fluorescence color of calcium phosphate tribasic (CPT) and a mixture of CPT and collagen I elicited by Lac dye (LD) CPT does not autofluoresce (*arrow* in **A**) but exhibited *red fluorescence* in the presence of LD (*arrow* in **A1**). A mixture of collagen I and CPT does not autofluoresce (*arrow* in **B**), but exhibits *purple fluorescence* in the presence of LD (*arrow* in **B1**)

LD did not autofluoresce when fluorescent light was excited at 345 ± 15 nm and emitted at 420 nm. When LD was mixed with CPT, a red fluorescence was elicited but not by other calcium compounds. Collagen fibers, which are mostly composed of collagen I and are abundantly co-exists with other substances in coronary plaques, exhibited blue fluorescence. When LD was added to a mixture of CPT and collagen I, a purple fluorescence was elicited (Fig. 2).

The other calcium compounds and the major substances that constitute atherosclerotic plaques did not exhibit purple fluorescence in the presence of LD, indicating this fluorescent color is characteristic of CPT only (Table 1).

**Table 1:** Autofluorescence and fluorescent color of the major substances comprising atherosclerotic plaques when excited by lac dye (LD) and imaged with color fluorescent microscopy

Color fluorescent microscopy		
Substances	Autofluorescence	Fluorescence in the presence of LD (10^− 5^ M)
Calcium phosphate tribasic	No	R
Calcium triphosphate tribasic + Collagen I	No	P
Hydroxyapatite (powder)	No	No
(Crystal)	W	No
Hydroxyapatite + collagen I	No	No
Other calcium compounds	No	No
High-density lipoprotein	No	No
Oxidized low-density Lipoprotein	No	No
Low-density lipoprotein	No	No
Very low-density lipoprotein	No	DBr
Lysophosphatidylcholine	No	No
Phosphatidylcholine	No	No
Triglyceride	No	No
Apolipoprotein B-100	No	No
Apolipoprotein A-1	No	No
Apolipoprotein E-2	No	No
Matrix metalloproteinase −1,−9	No	No
Cholesterol	Y	No
Cholesteryl oleate	No	No
Cholesteryl linoleate	No	No
7-Keto cholesterol	No	No
Oleic acid	No	No
Linoleic acid	No	No
Collagen I	B	LR
Collagen IV	LB	No
Collagen III, V	No	No
Heparan sulfate	No	No
Hyaluronic acid	No	No
Albumin	No	No
Globulins	No	No
Ceramide	Y	No
Elastin	LY	No
Hydroxyapatite	No	No
Proteoglycans	No	No
β-Carotene	O	No

*B* blue, *DBr* dark brown, *G* green, *LB* light-blue, *LR* light-red, *LY* light-yellow, O orange, *P* purple, *R* red, *W* white, *Y* yellow, *no* no fluorescence

LD (10^− 5^ M) was added to each substance to elicit color fluorescence

*Purple* fluorescence was evoked by adding LD to a mixture of CPT and collagen I. This fluorescence was not evoked in any other known substances that comprise atherosclerotic plaques listed in this table, indicating that this purple fluorescence is characteristic of only CPT

### CPT visualized by CFA

**Fig. 3:**
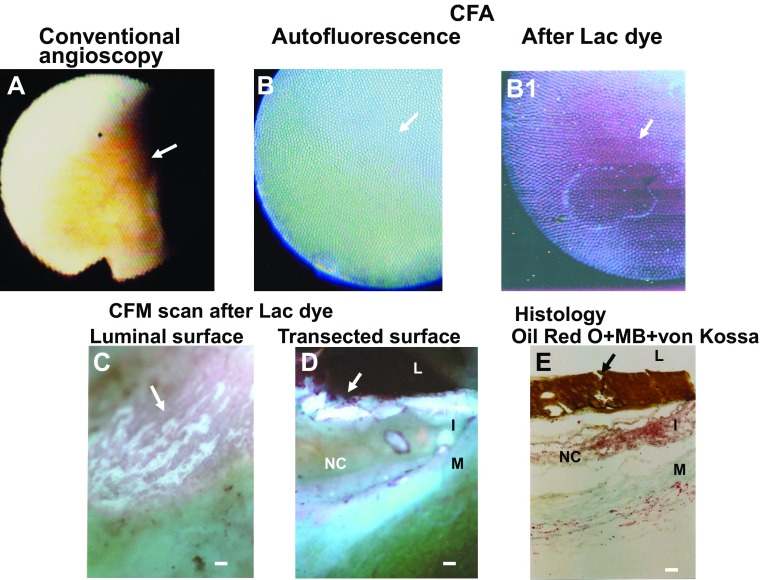
Color fluorescent angioscopic (CFA) and microscopic (CFM) observation of calcium phosphate tribasic (CPT) in a *yellow plaque* with a necrotic core (NC) The *yellow plaque* (*arrow* in **A**) exhibited diffuse *green autofluorescence* (*arrow* in **B**), indicating deposition of β-carotene with collagen I, lipids and/ or calcium compounds, which then exhibited *purple fluorescence* (*arrow* in **B1**) after the administration of Lac dye (LD), indicating the presence of CPT. *Purple fluorescence* was observed in luminal surface by CFM (*arrow* in **C**). The transected surface scan revealed that the CPT covered the plate-like structure that exhibited *white fluorescence* (*arrows* in **D**). The plate-like structure was stained *dark brown* by histology, strongly suggesting hydroxyapatite deposits (*arrow* in **E**). *L*, *I* and *M* indicate lumen, intima and media, respectively. *Bar* 100 µm

**Fig. 4:**
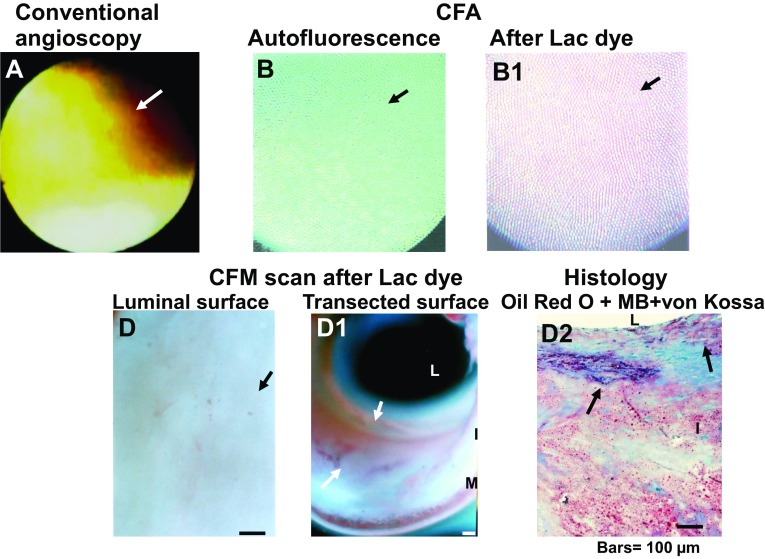
Color fluorescent angioscopic (CFA) and microscopic (CFM) visualization of calcium phosphate tribasic (CPT) in a *yellow plaque*. a *yellow plaque* (*arrow* in **A**) exhibited *light-yellow autofluorescence*, indicating the presence of collagen I and lipids (*arrow* in **B**) [7]. Plaque exhibited a *light-purple fluorescence* (*arrow* in **B1**) in the presence of Lac dye (LD), indicating the presence of CPT. Luminal and transected surface scanning by CFM revealed amorphous *light-purple* material (*arrows* in **D** and **D1**). The amorphous deposits corresponded to *amorphous purple* deposits visualized by histology (*arrow* in **D2**), indicating the presence of CPT. *L*, *I* and *M* indicate lumen, intima and media, respectively. *Bar* 100 µm

Before LD administration in the CFA studies, coronary luminal surface exhibited blue and green in mosaic fashion (Fig. 3B), or diffusely green autofluorescence (Fig. 4B). Blue fluorescence is the autofluorescence of collagen I and green autofluorescence indicates autofluorescence of a collagen I-β-carotene-lipid complex or a collagen β-carotene-calcium complex, respectively [7].

After LD administration, CPT showed patchy purple, diffuse dark-purple (Fig. 3B1) or diffuse-light purple fluorescence (Fig. 4B1). CPT exhibiting diffuse dark-purple fluorescence by CFA (Fig. 3B1) was revealed by CFM scan of the luminal and transected surfaces to be a superficial white plate-like structure covered by CPT (Fig. 3C, D). Histology revealed a dark-brown plate-like structure (Fig. 3D), indicating a plate-like hydroxyapatite.

CPT that exhibited diffuse light-purple fluorescence by CFA (Fig. 4B1), also exhibited amorphous light-purple fluorescence by CFM scan of the luminal and transected surfaces (Fig. 4D, D1), indicating the presence of amorphous CPT deposits. Histology revealed amorphous purple structures, indicating deposition of amorphous CPT co-deposited with collagen I (Fig. 4D1).

### Relationship between CPT deposition and plaque morphology

**Fig. 5:**
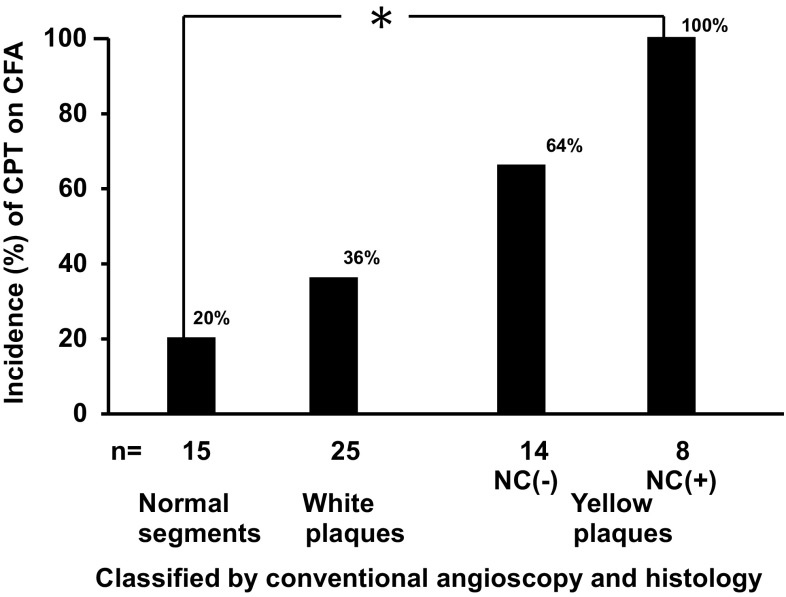
The relationship between the percentage (%) incidence of calcium phosphate tribasic (CPT) and plaque morphology. The incidence of CPT increased in the order of normal segments, white plaques, *yellow plaques* without necrotic core (NC) and *yellow plaques* with NC. *n* number of preparations examined

The incidence (%) of CPT visualized by CFA was low in normal coronary segments, showed a tendency to increase in white plaques and furthermore in yellow plaques. The incidence of CPT in yellow plaques with NC was significantly higher than in normal segments (Fig. 5).

CFA could detect 29 of the 46 CPT deposits that were visualized by CFM scan of the transected surface. The sensitivity of CFA was high in detecting CPT (irrespective of amorphous deposits or covering calcium crystals) deposited within 200 µm of the surface. The majority of CPT deposits which were not visualized by CFA were located in layers deeper than 200 µm.

## Discussion

Various invasive and non-invasive imaging techniques are used clinically to image coronary calcifications [13–18]. To date, however, imaging the individual calcium compounds in vivo including CPT, is beyond the scope of any of these imaging techniques.

Using a peptide, Cy-HABP-19 as an indicator, Lee et al. visualized hydroxyapatite in culture vascular smooth muscle cells [19]. Using Raman spectroscopy, Bonetti et al. observed hydroxyapatite in calcified human aortic valves or carotid plaques obtained by endoatherectomy [20]. To our knowledge, to date there are no reports in which native coronary CPT has been visualized ex vivo or in vivo.

### Fluorescent color of CPT

In the present CFM study, use of LD elicited red fluorescence of CPT and purple fluorescence in the presence of collagen I and CPT. On CFA, purple, but not red fluorescence was elicited by LD in excised coronary plaques. It is known collagen I exists even in the advanced stage of coronary plaques (i.e., yellow plaques with thin fibrous cap and NC) [7]. This may be the why purple fluorescence was elicited by LD in coronary plaques.

### Amorphous CPT

CPT showed an amorphous pattern of deposition, suggesting that CPT deposits in amorphous but not in crystal form in the vascular wall. CPT deposited alone or covered hydroxyapatite, suggesting that the former is before polymerization to hydroxyapatite and the latter in the process of hydroxyapatite growth. CPT which could be visualized by CFA. but It remains to be elucidated whether CPT is detectable by intravascular ultrasonography, optical coherence tomography or near-infrared spectroscopy, both of which are frequently used in the clinical setting.

### CPT deposition increased with plaque maturation

The incidence of CPT and of calcium crystals (hydroxyapatite) covered by CPT increased with plaque growth (white plaques) and further with plaque maturation (yellow plaques), suggesting that CPT participated in formation and growth of hydroxyapatite.

### Role of CPT in vascular calcification

Otsuka et al. reported that calcification often occurs in the presence of apoptosis of smooth muscle cells and macrophages with matrix vesicles accompanied by expression of osteogenic markers within the vessel wall [21]. Villa-Bellosta et al. reported that elevated serum phosphorous is a risk factor of vascular calcification [22]. Hortells et al. reported that amorphous calcium phosphate converts into hypdoxyapatite in cultured rat vascular smooth muscle cells [23]. Asaoka et al. reported that porous beads implant composed of CPT and hydroxyapatite becomes bone in which hydroxyapatite remains but CPT disappears, suggesting conversion of CPT into hydroxyapatite [24].

Although direct evidences which demonstrate conversion of CPT into hydroxyapatite are lacking, these reports and the similarity of chemical formulas, it is easy to consider that CPT [Ca_5_(OH)(PO_4_)_3_] is converted by polymerization into hydroxyapatite [Ca_5_(OH)(PO_4_)_3_]_x_. In the present study, CPT covered all calcium crystals (hydroxyapatite), irrespective of their size and location, a finding that supports the possibility that the overlying CPT was in the process of converting to hydroxyapatite.

### Prevention of vascular calcification

Phytate and phosphocitrate analogue inhibit hydroxyapatite formation in animal models but whether they act on CPT remains unknown [25–27]. Clinically applicable preventive therapy on vascular calcification does not currently exist. Based on the results of the present study, we consider that inhibition of the formation or conversion of CPT into hydroxyapatite could be a direct and effective preventive therapy for coronary and other vascular calcification.

### Study limitations

The present experimental study using LD as a biomarker of CPT appears to be the first to image native CPT in the human vascular wall. However, this study has some shortcomings. (1) Imaging was limited to target within 200 μm of the plaque surface and therefore deposits in the deeper layers could not be analyzed by the CFA system [3]. However, by improving the light source, light guide, image guide and camera, CPT deposited deeper than 200 μm would be visualized. (2) The number of autopsy cases in each disease group was very small, and therefore, it is not conclusive whether CPT deposition in coronary plaques is related to the underlying disease.

Nevertheless, CFA using CD could be used for analyzing the molecular mechanism(s) of coronary calcification and for discovery of a new preventive therapy for coronary calcification.

## Conclusions

We found procedure to visualize calcium phosphate tribasic (CPT) in human coronary artery, by CFA and CFM using LD as a biomarker. This procedure may be the first way to identify specific calcium compound and its localization in human coronary plaques and has a potential use in patients in vivo. The residual problem, clinical safety of intravascular administration of LD, remains to be clarified before clinical application.

## Electronic supplementary material

Below is the link to the electronic supplementary material.

Supplementary material 1 (PPT 34499 KB)

## Abbreviations

CFA: Color flurescent angioscopy
CFM: Color fluorescent microscopy
CPT: Calcium phosphate tribasic
LD: Lac dye
NC: Necrotic core
