# Supplementary material for: Fluorescent angioscopic imaging of calcium phosphate tribasic: precursor of hydroxyapatite, the major calcium deposit in human coronary plaques
Source: Int J Cardiovasc Imaging. 2017 Apr 21;33(10):1455–62. doi: 10.1007/s10554-017-1142-y (PMC5676831; doi:10.1007/s10554-017-1142-y)
Supplement: Supplementary file 1 — Supplementary material 1 (PPT 34499 KB) [file 10554_2017_1142_MOESM1_ESM.ppt]

## Slide 1
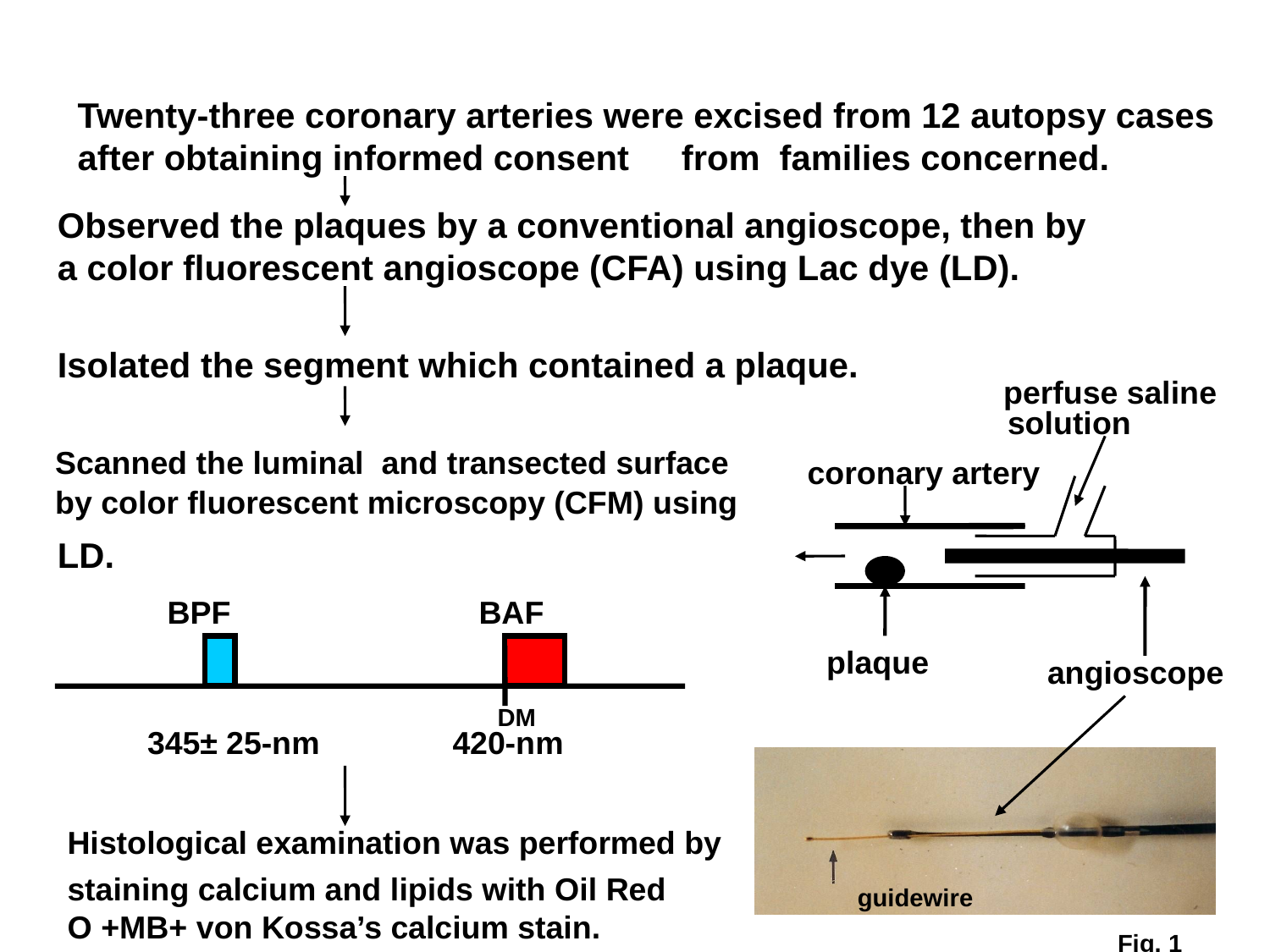

Twenty-three coronary arteries were excised from 12 autopsy cases after obtaining informed consent　from families concerned.
Observed the plaques by a conventional angioscope, then by a color fluorescent angioscope (CFA) using Lac dye (LD).
Isolated the segment which contained a plaque.
perfuse saline
solution
 Scanned the luminal and transected surface
coronary artery
 by color fluorescent microscopy (CFM) using
LD.
BPF BAF
plaque
angioscope
DM
345± 25-nm 420-nm
Histological examination was performed by
staining calcium and lipids with Oil Red O +MB+ von Kossa’s calcium stain.
guidewire
Fig. 1

## Slide 2
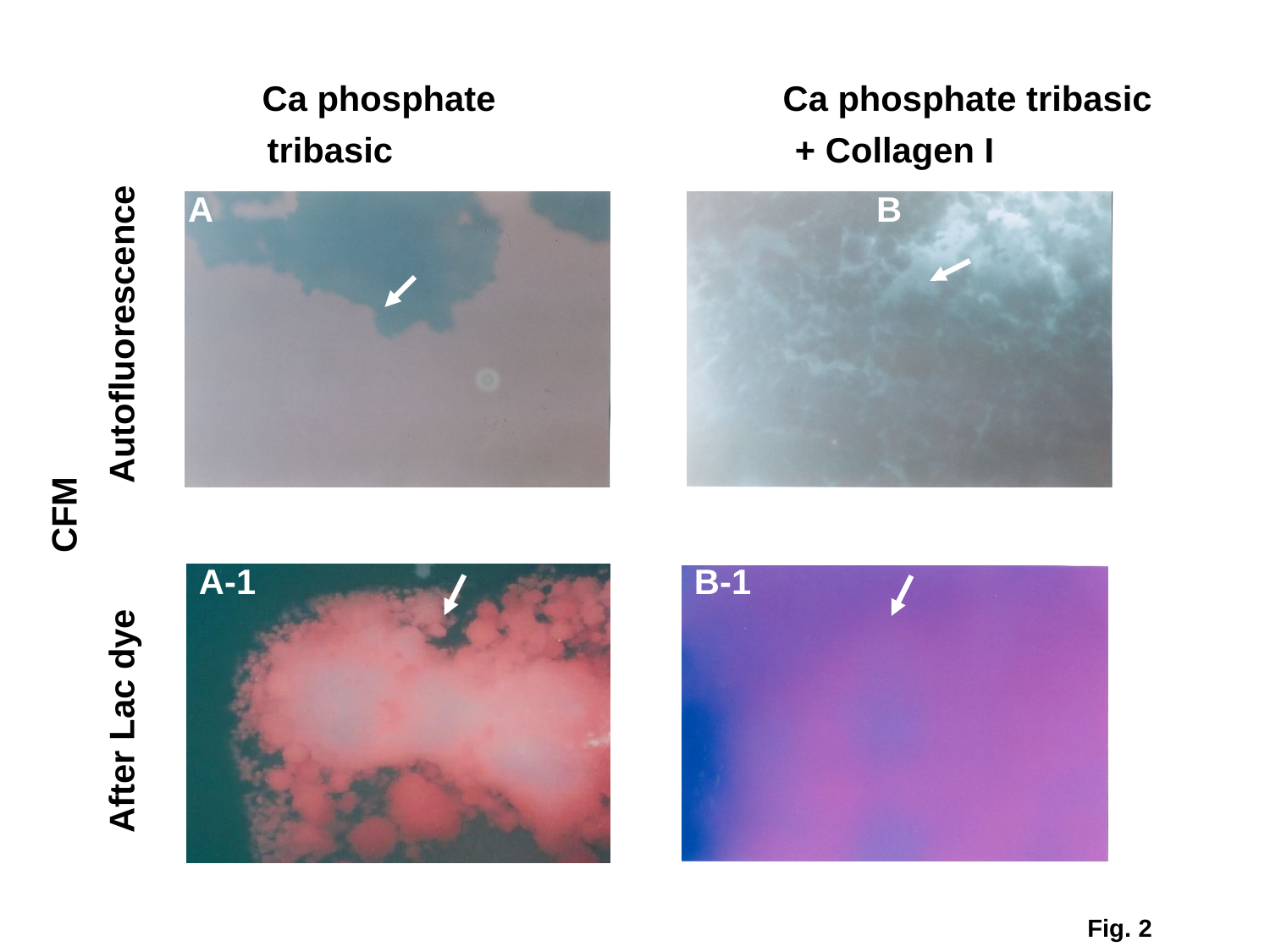

Ca phosphate 　　　 Ca phosphate tribasic
 tribasic 　　　　 + Collagen I
A 　　　　　　　 B
 After Lac dye Autofluorescence
CFM
B-1
A-1 B-1
Fig. 2

## Slide 3
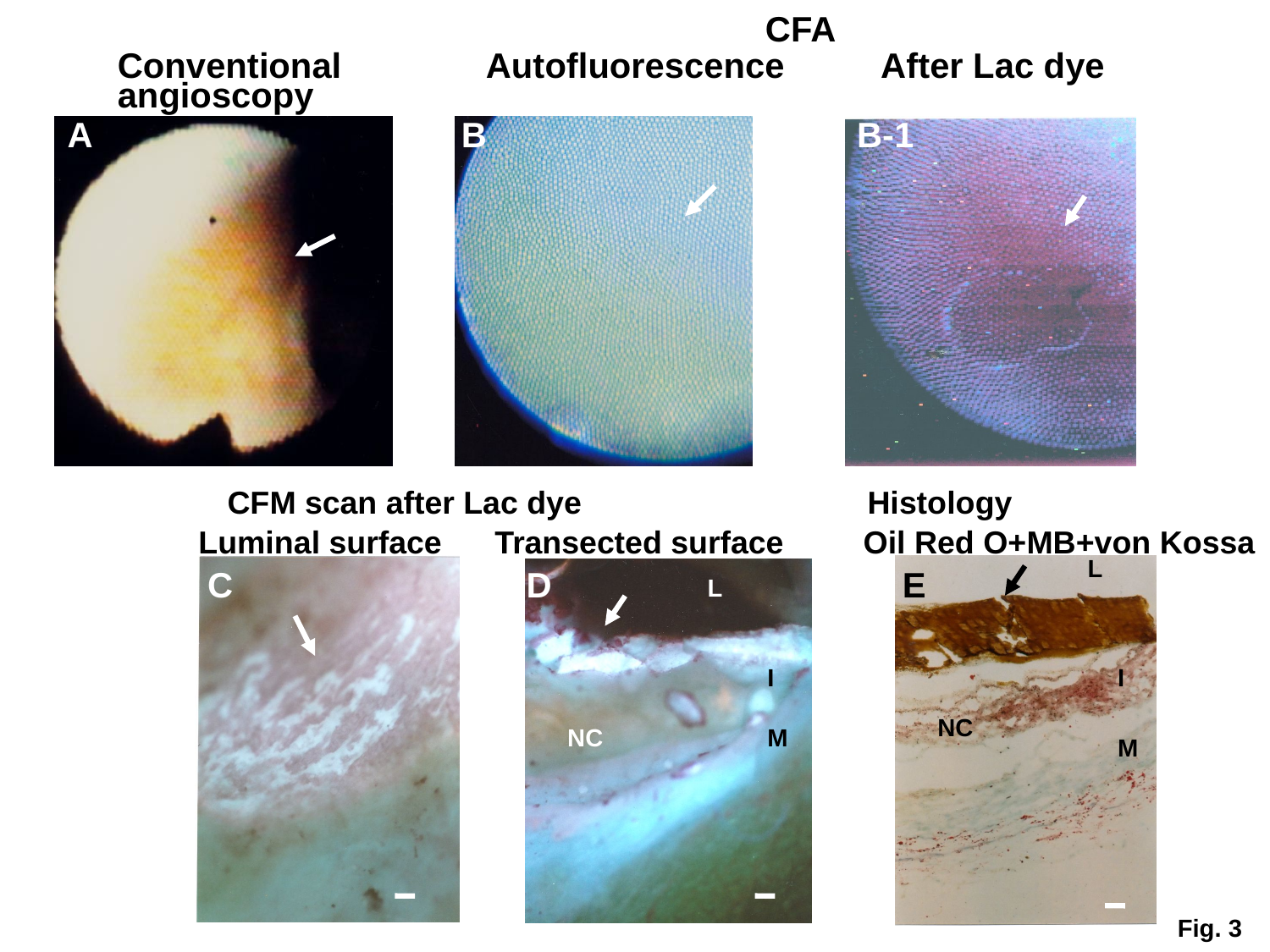

CFA
Conventional
 Autofluorescence After Lac dye
angioscopy
A B B-1
A
A B
CFM scan after Lac dye
Histology
 Luminal surface Transected surface Oil Red O+MB+von Kossa
L
 D E
C
L
I
I
NC
NC
M
M
Fig. 3

## Slide 4
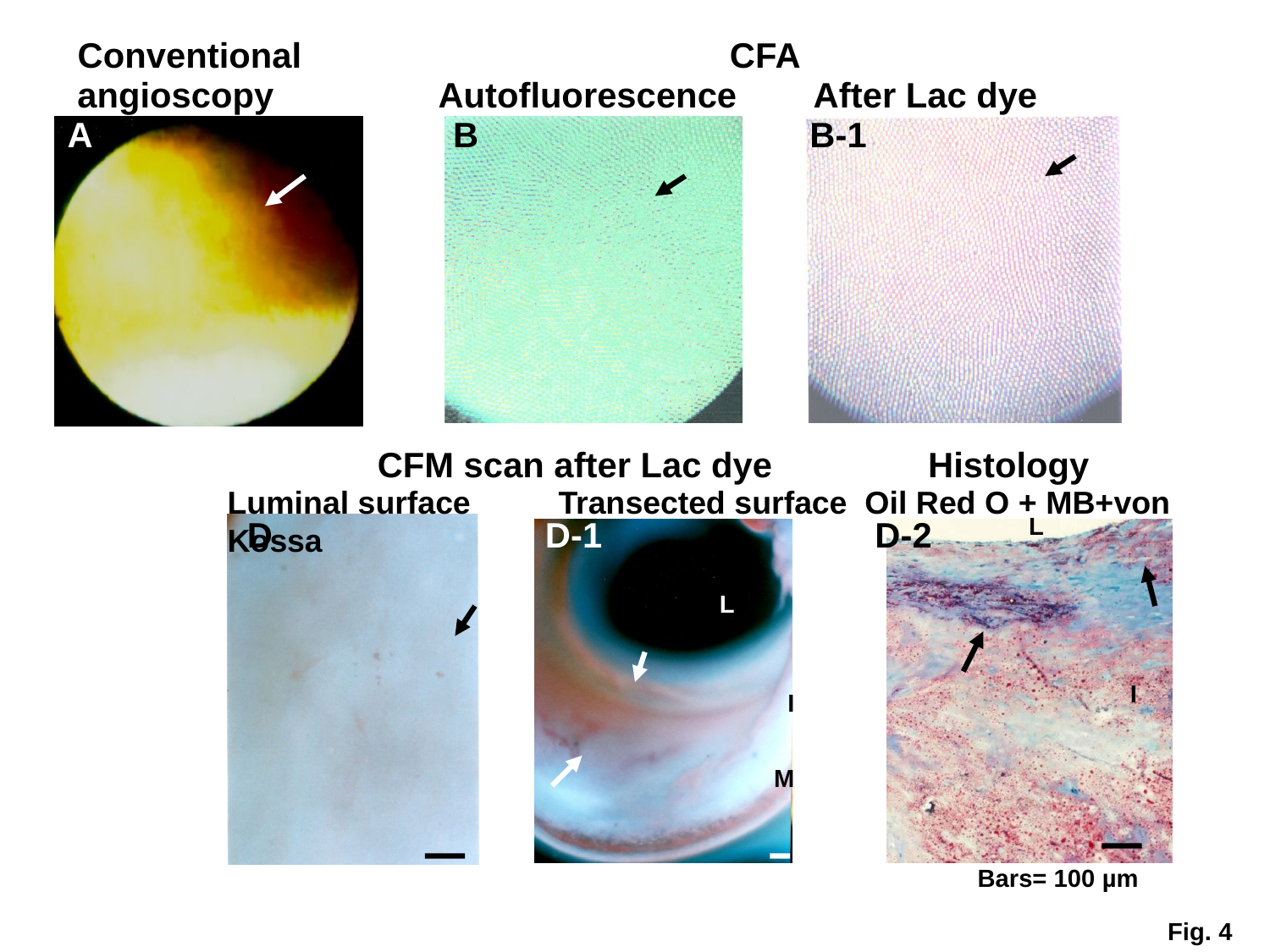

Conventional CFA
angioscopy Autofluorescence After Lac dye
A B B-1
CFM scan after Lac dye Histology
Luminal surface 　　Transected surface Oil Red O + MB+von Kossa
L
C D D-1 D-2
L
I
I
M
Bars= 100 µm
Fig. 4

## Slide 5
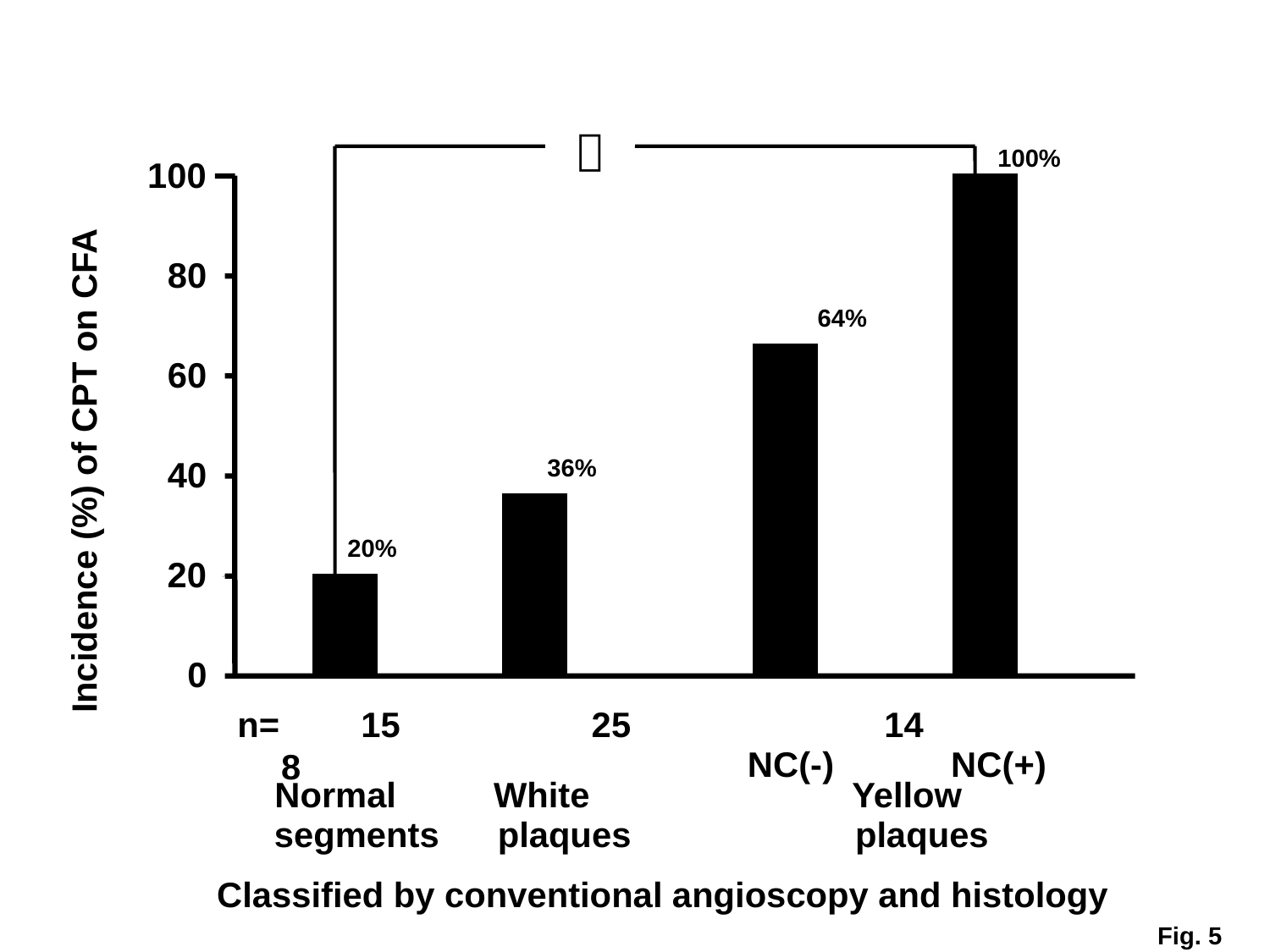

＊
100%
100
80
64%
60
Incidence (%) of CPT on CFA
40
36%
20%
20
0
n= 　15 　 25 　　14 　8
NC(-) NC(+)
 Normal White Yellow
 segments plaques plaques
 Classified by conventional angioscopy and histology
Fig. 5
